# Supplementary material for: Evaluation the Effect of Sonodynamic Therapy with 5-Aminolevulinic Acid and Sodium Fluorescein by Preclinical Animal Study
Source: Cancers (Basel). 2024 Jan 5;16(2):253. doi: 10.3390/cancers16020253 (PMC10813429; doi:10.3390/cancers16020253)

Table S1: Results of statistical analysis of the effects of fluorescein-SDT therapy on animals with subcutanous tumors. Statistical analysis was performed using Student's t test, *: $p < 0.05$, ** $p < 0.01$.

Fluorescein(16mg/Kg) +FUS (0.25、0.3、0.35MPa.duty10%.20min)							
	control	FL only	FUS 0.25MPa	FUS 0.3MPa	FUS 0.35MPa	FL+FUS 0.25MPa	FL+FUS 0.3MPa
FL only	0.0094**						
FUS 0.25MPa	0.4049	0.123					
FUS 0.3MPa	0.1521	0.3975	0.1231				
FUS 0.35MPa	0.1378	0.0794	0.388	0.1247			
FL+FUS 0.25MPa	0.0088**	0.0033**	0.0169*	0.0178*	0.0282*		
FL+FUS 0.3MPa	0.0057**	0.0136*	0.0429*	0.0396*	0.0195*	0.435	
FL+FUS 0.35MPa	0.0012**	0.0034**	0.0157*	0.0031**	0.0073**	0.295	0.1529

Figure S1. Body weight changes of fluorescein-SDT treated animals during the experimental period.

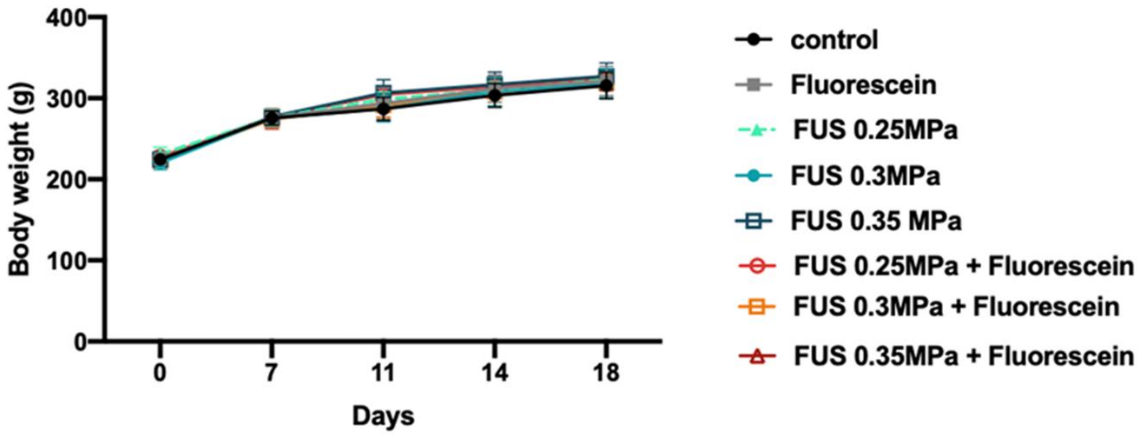

Figure S2. Cell uptake abilities and cytotoxic effects of 5-ALA on different type of cancer cells. Cancer cells were cocultured with 5-ALA for 6 hours, and cell viability was tested by CCK-8 assay. U87-MG: huamn malignant glioma; A549: human lung cancer; C6: rat glioma; A375: human melanoma; GL261, MBR614, MBR628, MBR323, MBR428: mouse glioma.

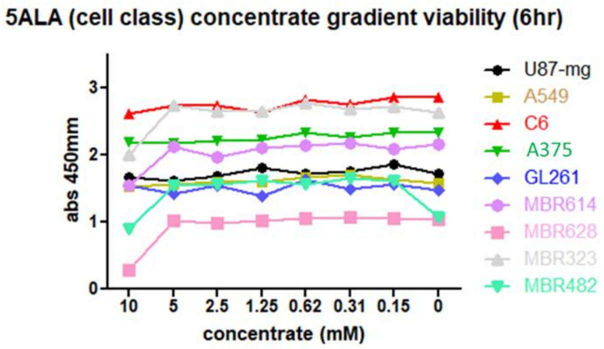

Figure S3. Human glioma cells U87-MG were implanted into Nu/Nu mice to evaluate the disruption of the blood-brain barrier (BBB) at early (9 days post-tumor implantation) and late stages (29 days post-tumor implantation). Evans Blue was intravenously injected through the tail vein into the mice. Evans Blue staining of the tumors indicates disruption of the BBB. Left: whole brain coronal sections, middle: the whole brain, right: MRI imaging of mice brain tumor.

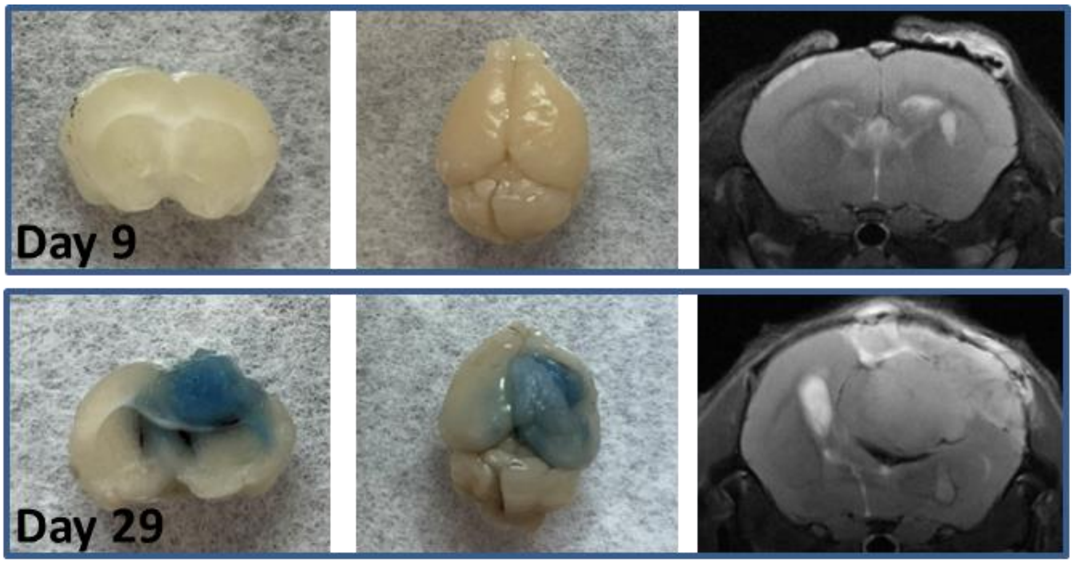

Supplement: Supplementary file 1 [file cancers-16-00253-s001.zip › cancers-2713873-supplementary.pdf]
